# Diffuse Interstitial Lung Disease Revealing Antisynthetase Syndrome

**DOI:** 10.7759/cureus.57513

**Published:** 2024-04-03

**Authors:** Hanane Benjelloun, Fatima Ezzahra Haouassia, Khadija Chaanoune, Nahid Zaghba, Najiba Yassine

**Affiliations:** 1 Pulmonology, Ibn Rochd University Hospital, Casablanca, MAR; 2 Genetics/Allergy, Ibn Rochd University Hospital, Casablanca, MAR; 3 Pulmonary Medicine, Ibn Rochd University Hospital, Casablanca, MAR

**Keywords:** antisynthetase syndrome, mechanics' hands, immunosuppressive therapy, anti-jo-1, interstitial lung disease

## Abstract

Interstitial lung disease (ILD) is a frequent manifestation of connective tissue diseases. They may be revelatory of the disease or occur during follow-up. Antisynthetase syndrome (ASS) is a complex and heterogeneous autoimmune disorder. Antisynthetase antibodies, in particular the anti-Jo-1 antibody, characterize this syndrome. The occurrence and severity of ILD determine the prognosis, which in turn determines therapeutic management. We report the case of a 53-year-old female patient presenting with ILD, revealing the diagnosis of ASS. The evolution was favorable with bolus corticosteroids associated with cyclophosphamide.

## Introduction

Antisynthetase syndrome (ASS) is an uncommon type of overlapping myositis that includes myositis, polyarthritis, Raynaud’s phenomenon, interstitial lung involvement, and fissured hyperkeratosis of the hands [1,2]. Antisynthetase antibodies, in particular the anti-Jo1 antibody, characterize this syndrome. The prevalence of interstitial lung disease (ILD) is higher with anti-Jo-1 antibody positivity [3]. The occurrence and severity of ILD influence the prognosis of ASS, which in turn influences the therapeutic management of ASS [4]. We describe a 53-year-old woman who had ILD linked to anti-Jo-1 antibodies and ASS.

The challenge of this situation comes from the fact that the diagnosis is made based on the deterioration of the patient’s symptoms, despite her use of corticosteroids and the presence of mechanic’s hands. To achieve a positive result, she required both bolus corticosteroids and cyclophosphamide. A better understanding of this respiratory disorder would facilitate prompt and suitable treatment, which would improve future prospects.

## Case presentation

**Figure 1:**
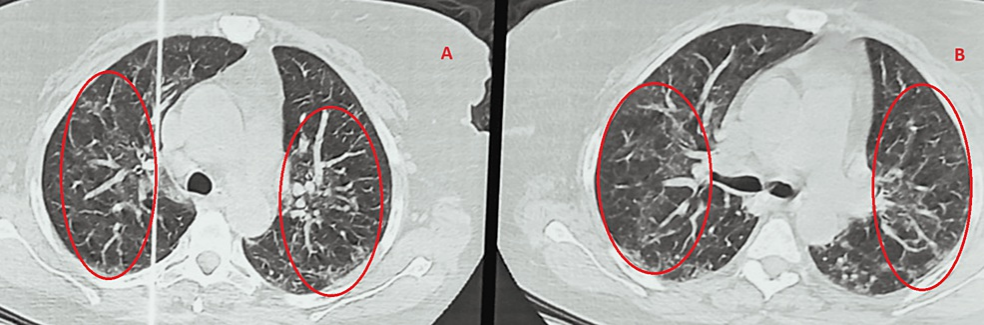
Chest CT scan performed in 2021 showing a bilateral frosted-glass image and septal thickening realizing the crazy paving appearance during her suspected COVID infection.

The patient was 53 years old, had type 2 diabetes, was taking oral anti-diabetics, had a history of wheezing respiratory issues that were diagnosed as asthma, and had received home treatment for a COVID infection without confirmation (based on highly suggestive clinical and scanographic evidence; Figure 1).

The patient came to see us six months later, at which point her dyspnea had progressed to stage 3 mMRC (Modified Medical Research Council), and she was experiencing a progressive onset of heaviness in her lower limbs more than her upper limbs. All of this was happening in the context of a little deterioration in overall health and fatigue. 

**Figure 2:**
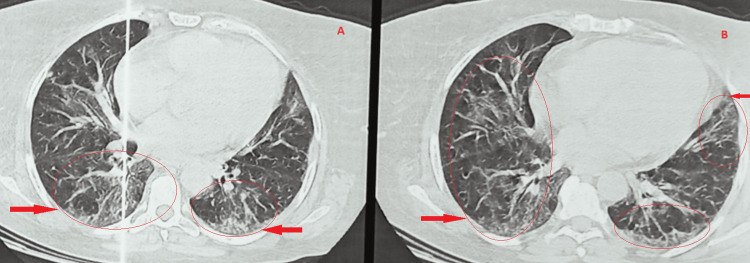
Chest CT scan performed during her hospitalization in 2022 showing a bilateral frosted-glass image and septal thickening realizing the crazy paving appearance.

During the clinical examination, the patient exhibited a respiratory rate of 20 breaths per minute, a heart rate of 87 beats per minute, and an oxygen saturation (SaO_2_) level of 94% in ambient air. Additionally, the patient had crackling sounds in the lower parts of the lungs. The neurological evaluation showed segmental strength scored at 4/5 in the lower limbs, without any additional anomalies. The thoracic computed tomography (CT) scan revealed an interstitial syndrome characterized by ground-glass regions mostly in the lower lobes and intralobular reticulations (Figure 2), and the blood count revealed a hyper-eosinophilia of 1850/mm^3^, which was observed and confirmed on two separate occasions with a 15-day interval.

Electroneuromyography (ENMG) that revealed evidence of axonal motor polyneuropathy in each of her four limbs was also beneficial. Pulmonary function tests revealed a somewhat restricted condition. The transthoracic cardiac echocardiogram showed no abnormalities. With wheezing, polyneuropathy, and hypereosinophilia, the diagnosis of eosinophilic granulomatosis with polyangiitis (Churg-Strauss) was suspected. The decision was taken to initiate full-dose corticosteroid medication and monitor her development.

The clinical and biological progress in this case was favorable, as evidenced by the normalization of the eosinophil count level. Nevertheless, when administered a dosage of 20 mg of corticosteroids, the patient’s neurological symptoms and difficulty breathing deteriorated.

**Figure 3:**
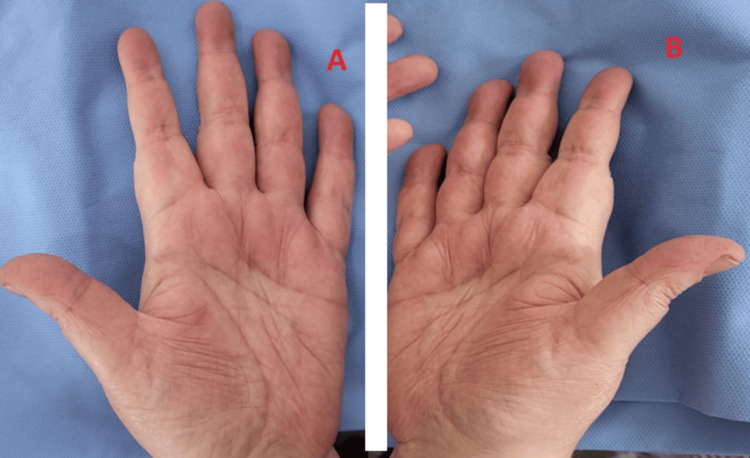
Patient's hands exhibiting cracks and fissures, resembling those commonly seen in mechanics (mechanic’s hands).

The patient had an oxygen saturation (SaO_2_) of 92% in free air during the clinical examination. The patient was tachypneic at 26 c/min and tachycardic at 102 b/min. She held the Barré but not the Mingazzini, and her neurological examination indicated a waddling gait. Her segmental strength in the lower limbs was assessed at 3/5 with no other anomalies. She also had crepitating rales in the pulmonary base. Both hands developed cracks, resembling the hands of mechanics (Figure 3).

**Figure 4:**
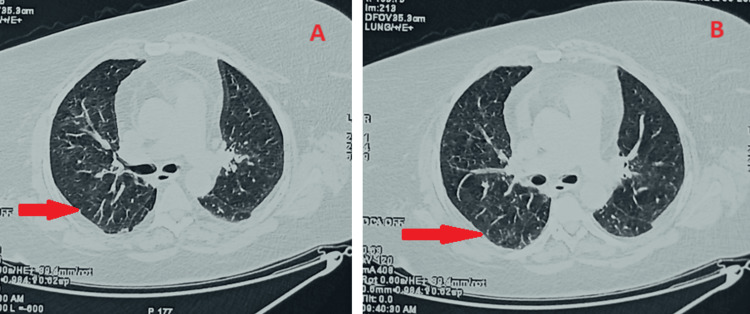
Chest CT images obtained after the patient underwent corticosteroid therapy revealed minor reticulations (indicated by red arrows) along with a noticeable reduction in frosted glass appearance.

A radiological clean-up was observed on a thoracic (CT) scan, with minor reticulations persisting despite a noticeable reduction of frosted glass (Figure 4).

Electromyography revealed extensive preganglionic injury in all three areas (areas of the cervical, thoracic, and lumbar spine), along with involvement of the anterior horn. The limbs’ motor potential was reduced in the upper limbs and collapsed in the lower limbs, and their sensitivity was preserved in all four limbs. Anti-Jo-1 antibodies were found to be strongly positive after a repeat immunological workup.

Her plethysmography revealed a mild restrictive ventilatory disorder with a total pulmonary capacity of 66%. Her gasometry revealed a type 1 respiratory insufficiency with a PaO_2_ of 72 mmHg, PCO_2_ of 44 mmHg, and PH of 7.427. Her echocardiography revealed no evidence of pulmonary hypertension (PH), with pulmonary arterial pressures estimated at 19 mmHg. These tests were performed as part of a follow-up assessment.

The diagnosis of antisynthetase antibodies was confirmed based on the presence of Jo-1 antibodies, interstitial involvement, neurological involvement, and the manifestation of mechanic’s hands as part of the dermatological involvement.

**Figure 5:**
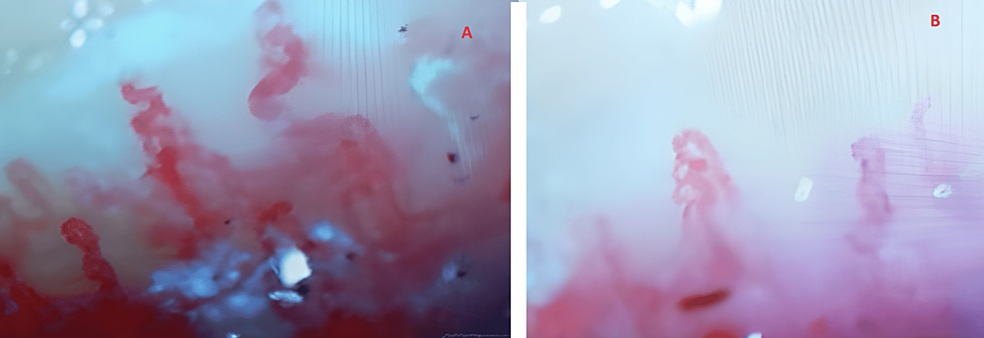
Capillaroscopy showing distal microangiopathy.

As a consequence, capillaroscopy showed a reduction in the quantity of capillaries, with the presence of abnormally enlarged capillaries and bleeding observed at one location. The presence of these abnormalities was observed on all eight fingers (it spared both thumbs), resulting in the manifestation of severe distal microangiopathy (Figure 5).

The levels of creatine phosphokinase were within the normal range. The patient was administered one-gram boluses of Solumedrol in combination with one-gram monthly boluses of cyclophosphamide, resulting in a highly favorable clinical outcome.

## Discussion

Idiopathic inflammatory myopathies were first described by Peter and Bohan in 1975 [5]. They are marked by progressive weakness in the proximal muscles, elevated serum creatine kinase levels, specific electromyographic abnormalities, and inflammatory infiltrates in the skeletal muscle on biopsy. The study of autoantibodies associated with this pathological entity has made it possible to define and classify different clinical entities. In 1990, Marguerie et al. were the first to describe ASS as a primary inflammatory myositis that was often linked to ILD and marked by autoantibodies that target one of the tRNA synthetases [6].

The most frequent of these is the anti-Jo-1 antibody. Subsequently, seven other autoantibodies were discovered (anti-PL12, anti-PL7, anti-OJ, anti-EJ, anti-KS, anti-Zo, and anti-YRS). However, their mutual presence is exceptional. ASS accounts for 30-35% of inflammatory myositis, and its incidence remains rare, in line with that of inflammatory myopathies, currently estimated at 11/100,000 inhabitants [7].

ASS affects more women than men (sex ratio F/M:3:2), with no age predominance. Pulmonary involvement in ASS is found in 67% to 100% of cases [8]. Its pathophysiology remains unclear, but it seems that the lung is the organ where tRNA synthetase becomes abnormally immunogenic. This condition is more frequently associated with anti-PL7, anti-PL-12, and anti-KS-positive ASS [9].

ILD mainly takes three forms: acute ILD, subacute/chronic ILD, and asymptomatic interstitial disease diagnosed on chest CT [9]. ILD may precede the diagnosis of ASS in 18% of cases, be diagnosed simultaneously with ASS in 64% of cases, or appear during the course of ASS in 18% of cases [9].

High-resolution thoracic CT remains the key examination for the investigation of ILD associated with ASS. Spiral acquisition is performed at the end of deep inspiration, with the patient supine, using thin native slices (1-2 mm). Reading is performed with double windowing of the parenchymal and mediastinal sections. For better micronodule detection, multiplanar reconstructions and the maximum intensity projection (MIP) mode are used to find the apico-basal gradient and the MIP mode. Along with ground-glass attenuation, intra-lobular reticulations, condensation, honeycomb, pulmonary nodules, and micronodules, thickening of interlobular septa and nonseptal lines, and traction bronchiectasis [10], these are the main abnormalities that are found.

In our patient, interstitial involvement was indicative of the diagnosis of ASS. The diagnosis of ASS was made on the basis of the combination of IDP, neurological and dermatological (mechanic’s hands) involvement, and anti-Jo-1 antibody positivity. Indeed, the presence of anti-tRNA synthetase antibodies signals the diagnosis in the presence of a suggestive clinical and radiological picture. The most common autoantibodies are anti-Jo-1, anti-PL7, and anti-PL12 [9]. In our case, no muscular involvement was detected. The use of electromyograms, magnetic resonance imaging, or histology is not systematic [9], but in our case, ENMG was performed in view of the patient’s symptoms.

Bronchial fibroscopy with bronchioloalveolar lavage is often done and gives specific results, such as lymphocytic alveolitis, usually CD8, or neutrophilic alveolitis, which is sometimes linked to eosinophilia [7]. Histology is not mandatory for diagnosis [4]. On a scan, the two most common basic lesions in ASS-associated ILD are ground-glass, which shows up in 80% of cases, and reticulations, which show up in 74%. There is a clear predominance of lesions in the basal and subpleural regions [10]. The radiological features most associated with ASS are nonspecific ILD in 59% of patients, common ILD in 23%, and organized lung disease in 17% [10].

Functional respiratory examinations classically show a restrictive ventilatory disorder with a decrease in local carbon monoxide diffusion. They not only help to orient the diagnosis but also to assess severity and monitor patients. The restrictive syndrome may be exacerbated by myositis-induced damage to the respiratory musculature [11].

The immunosuppressants most frequently prescribed are cyclophosphamide, azathioprine, mycophenolatemofetil, ciclosporin, and tacrolimus [12]. No study has demonstrated the superiority of any of these in the context of ASS [4]. Immunoglobulin infusions are indicated in cases of swallowing disorders and/or inhalation pneumonitis [13]. Furthermore, in a limited number of refractory cases, promising results have been obtained with anti-CD20 antibodies (Rituximab).

However, there is no comparison with other available immunosuppressive therapies. In our patient, clinical and radiological improvement was observed with cyclophosphamide. Up-to-date vaccinations, including influenza, Haemophilus, and pneumococcal vaccinations, are strongly recommended, immunosuppressive treatments permitting. Pulmonary rehabilitation and muscle therapy are also beneficial.

## Conclusions

ILD is prevalent in individuals with ASS. The presence of anti-tRNA synthetase antibodies and distinct symptoms outside of the chest serve to confirm the diagnosis. The use of both corticosteroids and immunosuppressants should be considered due to the graveness of the prognosis and the inadequate response to corticosteroids alone in these patients. Besides conventional diagnostic and follow-up examinations, respiratory function tests offer pertinent data for evaluating the effectiveness of treatment. Testing for anti-synthetase antibodies is currently recommended for all individuals with undiagnosed inflammatory diffuse polymyositis.
